# Epidemiology of Health Effects of Radiofrequency Exposure

**DOI:** 10.1289/ehp.7306

**Published:** 2004-09-23

**Authors:** ICNIRP (International Commission for Non-Ionizing Radiation Protection) Standing Committee on Epidemiology:, Anders Ahlbom, Adele Green, Leeka Kheifets, David Savitz, Anthony Swerdlow

**Affiliations:** ^1^Institute of Environmental Medicine, Karolinska Institutet, Stockholm, Sweden; ^2^Stockholm Center for Public Health, Stockholm, Sweden; ^3^Epidemiology and Public Health Unit, Queensland Institute of Medical Research, Brisbane, Australia; ^4^Department of Epidemiology, School of Public Health, University of California at Los Angeles, Los Angeles, California, USA; ^5^Department of Epidemiology, School of Public Health, University of North Carolina at Chapel Hill, Chapel Hill, North Carolina, USA; ^6^Section of Epidemiology, Institute of Cancer Research, Sutton, Surrey, United Kingdom

**Keywords:** electromagnetic fields, EMF, epidemiology, health effects, radiofrequency, RF

## Abstract

We have undertaken a comprehensive review of epidemiologic studies about the effects of radiofrequency fields (RFs) on human health in order to summarize the current state of knowledge, explain the methodologic issues that are involved, and aid in the planning of future studies. There have been a large number of occupational studies over several decades, particularly on cancer, cardiovascular disease, adverse reproductive outcome, and cataract, in relation to RF exposure. More recently, there have been studies of residential exposure, mainly from radio and television transmitters, and especially focusing on leukemia. There have also been studies of mobile telephone users, particularly on brain tumors and less often on other cancers and on symptoms. Results of these studies to date give no consistent or convincing evidence of a causal relation between RF exposure and any adverse health effect. On the other hand, the studies have too many deficiencies to rule out an association. A key concern across all studies is the quality of assessment of RF exposure. Despite the ubiquity of new technologies using RFs, little is known about population exposure from RF sources and even less about the relative importance of different sources. Other cautions are that mobile phone studies to date have been able to address only relatively short lag periods, that almost no data are available on the consequences of childhood exposure, and that published data largely concentrate on a small number of outcomes, especially brain tumor and leukemia.

The advent of mobile telephones, now used by about 1.6 billion people worldwide, has been accompanied by an upsurge in public and media concern about the possible hazards of this new technology, and specifically of radiofrequency field (RF) exposure. Although some epidemiologic research was conducted several decades ago on RFs in occupational settings, in general the effects of RFs in humans are an emerging area of investigation, and most studies are recent or not yet published. Furthermore, although the results of studies of mobile phone risks have received widespread public attention, their interpretation is not straightforward because of methodologic difficulties. In particular, because RFs are invisible and imperceptible, individuals cannot directly report on their exposure, and therefore the quality of exposure assessment needs particularly careful consideration when interpreting epidemiologic studies. In order to summarize the current state of knowledge, to explain the methodologic issues that need to be considered when assessing studies, and to aid in planning future studies, we have undertaken a broad review of epidemiologic knowledge about the effects of RFs on human health. We have divided the literature, for this purpose, into studies of RF exposure from occupational sources, from transmitters, and from mobile phones.

In this review we cover the possible effects of long-term exposure to RFs—defined as 100 kHz to 300 GHz—on the risk of diseases, for instance, cancer, heart disease, and adverse outcomes of pregnancy. We have not reviewed the health consequences of communications technology that are indirect or unlikely to be due to radiation. In particular, RFs can interfere with implanted medical devices, such as cardiac pacemakers, but the effects on health are a consequence of this interference, rather than a direct effect on the body; phone conversations by drivers of moving vehicles appear to raise the risk of motor vehicle accidents, but this is probably related to distraction rather than to RF exposure. Although anxieties and psychosomatic illnesses might be caused by knowledge of the presence of phones or phone masts, again, this would not be an effect of RFs and is not discussed.

As well as epidemiologic studies of disease causation, some studies have been published that use an epidemiologic design to investigate whether mobile phones can affect acute symptoms, such as headaches. For completeness, we have included these in this review, although such investigations are usually better conducted by laboratory volunteer experiments rather than by observational epidemiology, given the high degree of susceptibility to biased reporting in response to concerns.

Because this is primarily an epidemiologic review, we have not detailed the physics and dosimetry of RFs from different sources, which are described elsewhere [Hitchcock and Patterson 1995; Independent Expert Group on Mobile Phones (IEGMP) 2000; Mantiply et al. 1997]. However, because understanding of mobile-phone–related epidemiology is critically dependent on understanding of mobile phone technology, we have included some information explaining this technology. We have also included, because of its importance to future research advance, some comments on the interface between physics and epidemiology, and the gaps to be bridged between these disciplines if more rigorous investigation of potential RF effects is to be achieved.

## Exposure

### Sources of Exposure

Communications sources have increased greatly in recent years, and there is continuing change in the frequencies used and variety of applications. The first mobile phone systems were analog and used 450 and 900 MHz. Digital systems, operating at somewhat higher frequencies (1,800–1,900 MHz) and using different modulation techniques, became prevalent in the early 1990s. Currently, the third-generation systems using the Universal Mobile Telecommunication System are being introduced, which will operate in the 1,900–2,200 MHz frequency range. Occupational RF exposures occur to workers engaged in a number of industrial processes, particularly when using dielectric heaters for wood lamination and the sealing of plastics and industrial induction heaters. Relatively high levels of exposure to RFs can occur to workers in the broadcasting, transport, and communications industries and in the military, when they work in close proximity to RF transmitting antennas and radar systems. Medical exposures can come from medical diathermy equipment to treat pain and inflammation, electrosurgical devices for cutting tissues, and diagnostic equipment such as magnetic resonance imaging.

### Distribution of Exposure in the Population

Despite the rapid growth of new technologies using RFs, little is known about population exposure from these and other RF sources and even less about the relative importance of different sources. In a typical house, non-occupational exposure could come from external sources, such as radio, television (TV), and mobile-phone base stations, as well as internal sources, such as a faulty microwave oven, in-house bases for cordless phones, or use of mobile phones.

Radio and TV transmitters have a large coverage area and therefore operate at relatively high power levels up to about 1 MW (Dahme 1999). Although these transmitters could generate fairly high fields at ground level, most are not located in heavily populated areas and do not lead to high exposure of the population.

Mobile-phone base stations are low-powered radio transmitters that communicate with users’ handsets. In early 2000, there were about 20,000 base stations in the United Kingdom and about 82,000 in the United States. Base stations can transmit power levels of ≥ 100 W (Schüz and Mann 2000). It is expected that the number of base stations will roughly double to accommodate new technology and a larger percentage of sites will have to be shared between operators, complicating exposure assessment. The power density levels inside a building can be from 1 to 100 times lower than outside, depending on the type of building construction (Schüz and Mann 2000). In addition, exposure can vary substantially within the building. For example, exposure was found to be about twice as high (and more variable) in the upper compared with the lower floors of a building (Anglesio et al. 2001). Driven by a typical pattern of use, the exposure from base stations shows a distinct diurnal pattern, characterized by lowest values during the night and by two maxima during the day, the first from 1000 hr to 1300 hr and the second from 1800 hr to 2200 hr (Silvi et al. 2001). There have been few and limited efforts to characterize population exposures; all of them have been small (usually areas around 10–20 base stations) (Anglesio et al. 2001; COST281 2001; Schüz and Mann 2000). The total power density from the base stations was slightly higher than, but comparable with, the background power density from all other RF sources combined.

Mobile phones operate at a typical power of 0.25 W. Analog systems operated at higher power levels than the newer digital systems. Similarly, older cordless phones operated to the analog standard, whereas modern ones operate to the digital with a transmitted power of a base around 0.09 W in a home but higher in a business setting. The actual exposure of the user depends on a number of factors such as characteristics of the phone, particularly the type and location of the antenna; the way the phone is handled; and most important, the adaptive power control, which may reduce the emitted power by orders of magnitude (up to a factor of 1,000). Factors that influence adaptive power control include distance from the base station, the frequency of handovers, and RF traffic conditions. Thus, the emitted power is higher in rural than in urban areas and when the user is moving (e.g., in a car). In areas where there is a great deal of phone use, phones may operate more than half of the time at the highest power levels. To compensate for the shielding effect of materials, power levels of phones are, on average, higher when a phone is used indoors than outdoors. RF absorption is maximal on the side of the head to which the phone is held, greatest close to the antenna, and decreases to less than one-tenth on the opposite side of the head (Dimbylow and Mann 1999).

In an occupational setting, higher exposures occur, albeit infrequently; for example, radar exposed workers in the U.S. Navy had potential for exposures > 100 mW/cm^2^ (Groves et al. 2002).

### Epidemiologic Considerations in Exposure Assessment

#### General.

In the absence of information on what biologic mechanism is relevant, it is unclear what aspect of exposure needs to be captured in epidemiologic studies. Because heating is the only known effect of RFs, most research has assumed that the metric of choice must be a function of the specific absorption rate (SAR). Metrics used in epidemiologic studies of other agents, such as cumulative exposure, average exposure over specific time intervals, and peak exposure, need to be considered. Given the uncertainty about the relevant interaction mechanism, the dose needs to be assessed not just as external field intensity but also as SAR for specific anatomical sites. Integrating exposure over time is further complicated by the fact that sources vary markedly over very brief time periods relative to the time periods of interest.

Epidemiologic studies thus far have relied on rather crude proxies for exposure, such as job title, proximity to a base station, or use of a mobile phone. Refinement of exposure assessment is critical to improved epidemiology. This requires a bridge between the rather disparate worlds of epidemiology and physics. Although it is of interest to know about sources of variation or uncertainty in general, the critical need in epidemiologic studies is to identify those variables that are most important in determining exposure levels and most amenable to capture within populations.

A key element in linking the complexity of the exposure sources and patterns with the needs of epidemiology is a meter that is capable of monitoring individual exposure. Such meters have now been developed [National Radiation Protection Board (NRPB) 2003].

Ideally, the dose, time pattern, and frequencies (wavelengths) of exposure from all key sources should be estimated for each individual in the study. Dose– and duration–response analyses are important to assessment of etiology but have often been absent in the existing literature (Swerdlow 1999). In addition, the possible lag period between exposure and disease manifestation needs to be considered. Handheld mobile phones were not used regularly until the 1990s. Thus, studies published to date have had little power to detect possible effects involving long induction periods or effects from long-term heavy exposure to mobile phones or base stations.

Methodologically, it would be desirable to conduct studies to clarify the relative contributions of different spheres of life. Such knowledge would allow epidemiologists to design studies that incorporate all important sources of RF exposure, or at least determine how much it matters that the occupational studies to date have taken no account of residential or mobile phone exposures and vice versa.

#### Occupational exposures.

Most occupational epidemiologic studies have based their exposure assessments simply on job titles and have included no measurements (Tables 1–4). It is possible that some jobs (e.g., radar operator) are adequate indicators of RF exposure. However, many job titles that have been previously considered to indicate exposure may provide a poor proxy for RF exposure.

In addition to improving exposure assessment in individual studies, there is the potential to develop job–exposure matrices, with the rows corresponding to relatively homogeneous groups with respect to RF exposure, defined by job title, perhaps specific work location, calendar time, and other recordable work history, and the columns corresponding to RF exposure metrics.

#### Transmitter exposures.

All published epidemiologic studies of transmitter exposures have based exposure assessment on distance from the transmitter. The relation between exposure and distance from the antenna is usually very complex, especially in urban areas. Close to the antenna, the field is very low because of the directional antenna characteristics. As one moves away, the field pattern can be complicated, with peaks and valleys in field intensity with increasing distance from the antenna.

Estimation of community exposure to RFs from transmitters may, however, be amenable to refinement. Geographic information systems allow for precise assignment of residence, topography, and some other likely determinants of exposure. Historical information on power output from the transmitters may well be available. This information combined with personal measurements may provide refined measures of exposure that can be applied retrospectively, with empirical validation.

#### Mobile phones exposures.

Studies on mobile phones have used the simple dichotomy of user versus nonuser, with some incorporating information on years of use, number of phone calls per day, and duration of calls. Some studies have separated analog and digital phone use. Few have included use of cordless phones, which also generate RFs but from which exposure pattern is different.

Ongoing studies are attempting to incorporate information on intensity of use, place of use, position of the telephone, type of telephone, and calendar period of use. Each of these extensions need to be evaluated, however, to determine *a*) whether they are truly an important determinant of exposure and *b*) whether they are amenable to accurate historical reconstruction through recall or some type of written record. There is little benefit in knowing that the intensity of exposure varies by a parameter that cannot be captured, or gathering relatively precise information about, say, model of mobile phone, if no useful exposure variable can be derived from it.

## Mechanisms

Heating of cells and tissues from RF exposure might have benign or adverse biologic effects. These effects, which reflect an imbalance in the amount of heat built up in the body and the effectiveness of mechanisms to remove it, can be due to either elevated temperatures or increased physiologic strain from attempts to remove the heat. Of particular concern for whole-body heating are effects in the elderly, people taking certain kinds of drugs, and the fetus and infant. Cardiovascular mortality, birth defects, and impaired ability to perform complex tasks are among the outcomes that have been associated with whole-body heating. The sensitivity of different tissues and cells to thermal damage from both localized and whole-body heating varies. The central nervous system, testis, and lens of the eye seem to be particularly sensitive, the last due to a limited capacity to dissipate heat rather than a greater sensitivity of its cells to heat-induced damage.

Laboratory studies suggest that adverse biologic effects can be caused by temperature rises in tissue that exceed 1°C above their normal temperatures (Goldstein et al. 2003). In addition to the absolute increase in temperature, duration of heating and thermoregulatory capacity of the body are important determinants of the harmful levels of tissue heating. High rates of physical activity and warm and humid environments will reduce tolerance to the additional heat loads.

There has been concern about possible carcinogenic effects of RFs below levels that cause detectably harmful heating. RFs are not sufficiently energetic to destabilize electron configurations within DNA molecules. Thus, there is no direct link between RF exposure and genotoxic effects such as DNA mutations, DNA strand breaks, or other genetic lesions. Experimental evidence from animal and laboratory studies at the cellular level confirms the lack of genotoxic effect of RFs (Krewski et al. 2001; Moulder et al. 1999). Similarly, an investigation in rodents did not find support for the suggestion that growth of tumors induced by other agents may be promoted by RFs from mobile phone signals (Imaida et al. 2001; Mason et al. 2001).

Repacholi et al. (1997) evaluated the effects of RFs on tumorigenesis in a moderately lymphoma-prone Eμ-*Pim1* oncogenetransgenic mouse line. Exposure was associated with a statistically significant 2.4-fold increase in the risk of developing lymphoma. Utteridge et al. (2002) recently repeated this study with a larger number of mice and with several refinements in the experimental design and did not demonstrate any difference in the incidence or type of lymphomas that developed between control and treated groups. Questions have been raised about the conduct and reporting of both studies and the inconsistency has not been resolved (Goldstein et al. 2003). Additionally, extrapolating the transgenic model to humans remains controversial.

## Outcomes

A particular public concern appears to be that the use of handheld mobile phones may be linked to the occurrence of malignant disease, especially brain cancer and, to a lesser extent, leukemia. Other tumors such as acoustic neuroma that occur in the head and neck region have also been investigated. Each of these conditions is rare. The incidence of malignant tumors of the brain in the general population is around 10–15 per 100,000 each year (Behin et al. 2003); the annual incidence of benign extracerebral tumors such as meningiomas is about 3 per 100,000, and benign tumors of the cranial nerves, such as acoustic neuromas, are rarer still. Because tumor incidence is so low, investigators have so far relied on case–control studies or, in a few instances, retrospective cohort studies. In addition, different tumor subtypes are likely to have different causes, as evidenced among brain tumors by the different molecular pathways leading to malignant astrocytomas on the one hand and benign meningiomas and acoustic neuromas on the other (Inskip et al. 1995). Similarly, there are a variety of types of leukemia, each probably with differences in causation, making it even more difficult to ascertain sufficient numbers of homogeneous tumors for study. Epidemiologic assessments have been further complicated because the environmental risk factors for malignant and benign brain tumors (Inskip et al. 1995), and hence potential confounders, are largely unknown beyond high-dose ionizing radiation. For leukemia (Petridou and Trichopoulos 2002), knowledge of potential confounders is greater but still limited. Other risk factors, besides ionizing radiation, include exposure to chemotherapy, cigarette smoking, and benzene, as well as constitutional chromosomal abnormalities among children in particular.

Available evidence suggests that induction of brain tumors occurs over decades after tumorigenic exposures early in life. Latency of tumors varies from months to years depending on how aggressive tumor growth is and the location of the tumor. Epidemiologic studies should therefore in principle allow for a lead time between potentially causal exposure and disease, although in the absence of biologic or epidemiologic evidence it is unclear what length this should be for potential RF effects.

Other chronic diseases such as cardiovascular disease, as well as symptoms, both acute and chronic, have been studied in relation to RF exposure. Headaches and other cranial discomforts including sensations of local warmth or heating, dizziness, visual disturbances, fatigue, and sleeplessness are the main symptoms reported by users of mobile phones. All of these are common symptoms in humans.

## Review of Studies on Occupational Exposure

### Cancer

Information on cancer risks in relation to occupational RF exposure comes largely from three types of epidemiologic study: cohort studies, investigating a wide range of cancer (and non-cancer) outcomes in groups with potential RF exposure (Tables 1 and 2); case–control studies of specific cancer sites, investigating occupational RFs as well as other exposures (Table 3); and analyses of routinely collected data sets on cancer incidence or mortality, in which risks of cancer have been assessed in relation to job title (Table 4). The most extensive literature addresses brain tumors and leukemia.

Considering study size, design, and likely quality of RF assessment, the most informative studies (Groves et al. 2002; Milham 1988; Morgan et al. 2000) provide little evidence of an association with either brain tumors or leukemia. The one possible exception was an increased risk of nonlymphocytic leukemia in radar-exposed navy veterans (Groves et al. 2002) restricted to only one of three highly exposed occupations (aviation electronics technicians), but this finding was divergent from that of an earlier study of U.S. naval personnel (Garland et al. 1990). Two U.S. case–control studies of brain tumor etiology have shown elevated odds ratios (ORs) of around 1.5 in relation to jobs believed to have RF exposure. However, the study by Thomas et al. (1987) was based on interviews with relatives of dead cases and hence was unable to identify exposure with much certainty. The other study (Grayson 1996) assessed exposures by a job–exposure matrix based on historical reports of incidents of exposure above permissible limits (10 mW/cm^2^). No clear or consistent trend was found in risk of brain tumor in relation to exposure score. A widely cited study of U.S. embassy staff in Moscow and their dependents with possible RF exposure was only published as a précis by a third party (Goldsmith 1995); this leaves the study methods unclear, but few brain tumors or leukemia occurred, and half were in dependents who lived outside the embassy.

A key concern across all these studies is the quality of assessment of RF exposure, including the question of whether it was truly present at all and, if so, for what proportion of the cohort. Although the published studies do not give consistent evidence for an increased leukemia or brain cancer risk, they cannot be counted as substantial evidence against a possible association. Most of the studies suffer from severe imprecision, with the cancers of greatest interest rarely found in cohort studies of modest size and the exposure of interest rarely found in geographically based case–control studies. The cohort studies generally lack data on other relevant exposures, including non-radio frequencies of radiation, as well as on RF exposures outside the workplace (e.g., mobile phones). The studies based on routine data are vulnerable to publication bias given the many data sets worldwide that could be used to address this issue. Several of these studies did not follow workers after they left the job of interest (Garland et al. 1990; Grayson 1996; Szmigielski 1996), with the potential for bias if individuals left employment because of health problems that later turned out to be due to cancer; this might especially be a problem for some types of brain tumor, which can be present for long periods before diagnosis. In addition, several studies have had substantial methodologic inadequacies—for instance, one study that found apparently increased risks for many different cancers used more sources of exposure information for cancer cases than for noncancer subjects and was analyzed improperly (Szmigielski et al. 2001).

#### Breast cancer.

Several studies have investigated the risk of breast cancer in relation to RF exposure. A cohort study of radio and telegraph operators in Norwegian merchant ships by Tynes et al. (1996) found a relative risk (RR) of breast cancer of 1.5 [95% confidence interval (CI), 1.1–2.0), based on 50 cases in women working in this occupation, and stronger for women ≥ 50 years of age [2.6 (95% CI, 1.3–5.5)]. An elevated RR found also for endometrial cancer suggests that reproductive and hormonal factors (for which full adjustment could not be made), not RFs, may have been responsible for the increased breast cancer risk. A large case–control study based on job titles from death certificates in the United States found no trend in risk of breast cancer in relation to probability or to level of occupational RF exposure (Cantor et al. 1995). A case–control study in the United States of men with breast cancer found an OR of 2.9 (95% CI, 0.8–10) in radio and communication workers (Demers et al. 1991), based on seven cases in exposed men, and with a low response rate in controls. A study of U.S. embassy personnel with potential RF exposure found two breast cancers, with 0.5 expected (Goldsmith 1995). Other studies of male (Groves et al. 2002) and female (Lagorio et al. 1997; Morgan et al. 2000) breast cancers, with few cases, did not report increased risks. The available data are insufficient to reach any conclusion on whether RF exposure is related to breast cancer risk, but the results of Tynes et al. (1996) do support continued evaluation of the possibility.

#### Testicular cancer.

Testicular cancer was considered in a U.S. case–control study (Hayes et al. 1990). A significantly increased risk was found for self-reported occupational exposure to microwave and other radio waves (OR = 3.1) but not for self-reported radar exposure or for radar or other microwave exposure assessed by an occupational hygienist based on job history. A cluster of testicular cancer (observed/expected ratio = 6.9) was reported in six police officers in Washington State (USA), who routinely used handheld traffic radar guns (Davis and Mostofi 1993). In a large U.S. Navy cohort with radar exposure, testicular cancer mortality was lower than expected [standardized mortality ratio (SMR) = 0.6 (95% CI, 0.2–1.4) in the group with potential for high exposure (Groves et al. 2002).

#### Ocular melanoma.

Ocular melanoma was associated with self-reported exposure to microwaves (excluding domestic microwave ovens) or radar [OR = 2.1 (95% CI, 1.1–4.0)] in a case–control study (Holly et al. 1996). Stang et al. (2001) found an increased risk of ocular melanoma in subjects with self-reported occupational exposure for at least 6 months and several hours per day to RFs (14% of cases, 10% of controls) and for occupational exposure several hours per day to radio sets [OR = 3.3 (95% CI, 1.2–9.2)]. There was no relation of risk to duration of this exposure, however, and risk was not increased for radar exposure [OR = 0.4 (95% CI, 0.0–2.6)]. The study was small and combined subjects from two different study designs.

#### Lung cancer.

A nested case–control study of electrical utility workers in Quebec (Canada) and France thought to be exposed to pulsed electromagnetic fields found a significant excess of lung cancer (Armstrong et al. 1994) and a dose–response gradient with increasing cumulative exposure. Adjustment for crude indicators of smoking and other factors left the results little changed. In an attempt to address a similar exposure in a cohort of U.S. electric utility workers, limited because of the ill-defined agent addressed in the original study, no increased risk of lung cancer was found (Savitz et al. 1997). No other studies of RFs have reported associations with lung cancer (Groves et al. 2002; Lagorio et al. 1997; Milham 1985, 1988; Morgan et al. 2000; Muhm 1992; Szmigielski 1996; Szmigielski et al. 2001; Tynes et al. 1996).

In conclusion, there is no cancer site for which there is consistent evidence, or even an individual study providing strong evidence, that occupational exposure to RFs affects risk. The quality of information on exposure has generally been poor, however, and it is not clear that the heterogeneous exposures studied should be combined in etiologic studies. This, combined with imprecision and methodologic limitations, leave unresolved the possibility of an association between occupational RFs and cancer.

### Other Outcomes

#### Adverse reproductive outcomes.

A wide range of potential reproductive consequences of RF exposure have been investigated (Table 5), with a focus on exposures of physiotherapists to therapeutic short wave diathermy (typically 27.12 MHz). Depending on the type of equipment used and the location of the operator in relation to the equipment, substantial peak exposures can occur (Larsen and Skotte 1991). Many of the studies analyzed levels of exposure, on the basis of duration of work and type of equipment used (shortwaves or microwaves).

There are isolated suggestions of an association between RF exposure and delayed conception (Larsen et al. 1991), spontaneous abortion (Ouellet-Hellstrom and Stewart 1993; Taskinen et al. 1990), stillbirth (Larsen et al. 1991), preterm birth after exposure of fathers (Larsen et al. 1991), birth defects in aggregate (Larsen 1991), and increased male-to-female sex ratio (Larsen et al. 1991). Almost always, however, either the finding was not corroborated in other studies of comparable quality, or there are no other studies available. The evidence is strongest for spontaneous abortion (based on two independent studies with some support). Potential confounding by other aspects of work activity (e.g., physical exertion) needs to be considered, however.

Semen parameters have been examined among men with varying forms of military exposure to microwaves and radar (Table 5). Three of these studies found reductions in sperm density (Hjollund et al. 1997; Lancranjan et al. 1975; Weyandt et al. 1996), with variable results for other semen parameters. Several of these reports were based purely on volunteers, with no attempt to sample from a defined population (Lancranjan et al. 1975; Schrader et al. 1998; Weyandt et al. 1996), and those that did provide information about response proportions (Grajewski et al. 2000; Hjollund et al. 1997) had substantial nonresponse. However, given the well-known susceptibility of spermatogenesis to even subtle heating, the possibility of reduced fertility in exposed men is reasonable to evaluate.

Overall, problems of exposure assessment temper any conclusions regarding reproductive outcomes, and no adverse effects of RFs have been substantiated.

#### Cardiovascular disease.

Several methodologically weak studies from the Soviet Union addressed microwave exposure and acute effects on cardiovascular physiology (e.g., hypotension, bradycardia, tachycardia) as part of a set of ill-defined conditions (Jauchem 1997). Additional studies of considered symptoms among a range of potentially exposed groups including radar workers, pilots, radio broadcasting workers, and electronics industry workers. The variability in research methods, exposure characteristics, and outcome measures makes it difficult to draw conclusions: there are sporadic reports of symptoms among some groups of workers, but no obvious pattern is present.

Major clinical outcomes have been examined less frequently. In a mail survey of U.S. physical therapists (Hamburger et al. 1983) men more highly exposed to microwave and shortwave radiation, based on indices including length of employment and frequency of treatments, tended to report a significantly greater prevalence of heart disease, with ORs of 2–3. Selective response to this survey must be considered among possible explanations for the associations that were observed. In U.S. Navy veterans potentially exposed to radar (Groves et al. 2002) and in a cohort of nearly 200,000 Motorola workers (Morgan et al. 2000), heart disease SMRs were well below 1.0, and analyses of mortality (Groves et al. 2002), hospital admissions, and disability compensation (Robinette et al. 1980) did not support greater risk with greater potential exposure. Other cohort studies reporting cardiovascular mortality have had small numbers (Lagorio et al. 1997; Muhm 1992).

Overall, the literature on RFs and cardiovascular symptoms and disease provides little suggestion of an association but is at too rudimentary a level to draw firm conclusions.

#### Cataracts.

Laboratory research indicates that the lens of the eye is highly sensitive to heat, and damage can occur from even a single acute exposure. Hence, there is a potential mechanism for RFs to lead to increased cataract incidence. Epidemiologic research has been limited, however, especially with regard to exposure assessment.

Based on hospital records of U.S. military veterans (Cleary et al. 1965), men with cataracts were no more likely than men with other medical conditions to have been radar workers (OR = 0.67, *p* > 0.10). Age was adjusted using broad groupings, with little change to the result.

In two studies in the U.S. military, ocular examinations were conducted on microwave-exposed and unexposed workers, without knowledge of exposure status by the examiner. In one (Cleary and Pasternack 1966) a tendency toward increased minor lens changes was found among exposed workers, characterized as the equivalent of 5 years of advanced aging in the exposed compared with unexposed workers around 60 years of age. In the other (Shacklett et al. 1975), prevalence of lens opacities was similar in exposed and unexposed individuals matched on age.

In an Australian study of workers who built and maintained radio and TV transmitters, compared with unexposed workers from the same geographic regions (Hollows and Douglas 1984), posterior subcapsular opacities were in excess in exposed workers (borderline significant), but nuclear sclerosis prevalence was similar in exposed and unexposed workers. It was not specified whether evaluators were aware of exposure history. Exposures were estimated to be from 0.08 to 3,956 mW/cm^2^, with brief, intense exposures thought to be quite common.

The study designs above are limited with respect to exposure assessment and selection of unexposed workers. Solar radiation exposure, a known risk factor for cataracts, was not considered and could have differed between RF-exposed and unexposed workers. Not all of the opacities were of direct clinical importance, but they would be pertinent to a pathway that could lead to cataract later in life. The plausibility of a causal relation supports more extensive investigation.

## Review of Studies on Environmental Exposure from Transmitters

The primary concern with transmitters has been with cancer risk among populations who live in proximity to transmitters, including those that are used for transmitting radio, television, microwave, and cellular telephone communications. There is a long history of public concern and resistance to the siting of such antennas, for reasons involving aesthetics and property values, as well as health concerns. Much of the research has been conducted in response to such concerns, either based solely on the exposure source or based on a perceived cancer cluster among persons living in the vicinity.

The studies of which we are aware are listed in Table 6, together with some fundamental characteristics and major findings.

The first study (Selvin et al. 1992) in San Francisco, California (USA) was focused on statistical analysis of spatial data and the results are not reported according to standard epidemiologic practice and do not include RR estimates. The source of exposure was a large TV antenna, and the three statistical methods considered in the report all showed that the pattern of cancer incidence was essentially random with respect to the antenna. A case–control study based on an apparent cluster of childhood leukemia (Maskarinec et al. 1994) was prompted by an observation of an unusually large number of childhood leukemia cases in a region of Hawaii (USA). There were 12 leukemia cases, and the OR for having lived within 2.6 miles of the radio antennas before diagnosis was 2.0 (95% CI, 0.06–8.3). Hocking et al. (1996) compared cancer incidence in three municipalities immediately surrounding three TV transmitters in northern Sydney, Australia, with the cancer incidence in six adjacent municipalities, estimating power densities from information on commencement of service of each transmitter, power, and frequency band. For leukemia incidence in adults, they found an RR of 1.2 (95% CI, 1.1–1.4) for the inner three municipalities compared with the surrounding municipalities. Their highest RR, 1.7 (95% CI, 1.1–2.5), was for the subcategory “other leukemia.” For childhood leukemia, they observed an RR of 1.6 (95% CI, 1.1–2.3). Neither for adults nor for children were there any risk elevations for brain tumor.

Dolk et al. (1997b) reported on an apparent cluster of leukemia and lymphomas near a U.K. radio and TV transmitter at Sutton Coldfield. The study area was defined as a 10 km radius circle around the transmitter. Ten bands of increasing distance from the antenna were defined as the basis of testing for declining incidence with increasing distance. The RR of adult leukemia within 2 km was 1.8 (95% CI, 1.2–2.7), and there was a statistically significant decline in risk with increasing distance from the antenna. In children younger than 15 years of age, there were two cases compared with 1.1 expected within the 2 km radius circle. The authors concluded that there was an excess risk of adult leukemia in the vicinity of the transmitter.

A second investigation (Dolk et al. 1997a), with a design similar to that of the first one, was extended to include 20 high-power TV and FM radio transmitters. Inside the 2 km radius circle the observed:expected ratio for adult leukemia was 0.97 (95% CI, 0.78–1.2), and for childhood leukemia, 1.1 (95% CI, 0.61–2.1). Thus, these results gave no more than very weak support to the original results.

McKenzie et al. (1998) reexamined the Sydney results discussed above. They found that the excess risk reported by Hocking et al. (1996) was mainly limited to one local government area within the studied region.

The Sutton Coldfield results have also been followed up by another group (Cooper et al. 2001). They used more recent cancer data to reanalyze cancer incidence around the transmitter and found considerably weaker results than the original.

An Italian study occasioned by local concerns investigated leukemia incidence in children and leukemia mortality in adults within a 10 km circle around the Vatican radio station (Michelozzi et al. 2002). The station consists of numerous transmitters with different transmission powers ranging from 5 to 600 kW and with different frequency ranges. In adults of both sexes taken together, the SMR within 2 km of the station was 1.8 (95% CI, 0.3–5.5) based on two cases. Stone’s test for trend in rates over successive 2-km bands around the station gave a *p*-value of 0.14. The excess risk and the trend were essentially confined to males. In children, the standardized incidence ratio (SIR) for those living within the 2 km radius circle was 6.1 (95% CI, 0.40–28) based on one case. Elevated rates were observed for all cumulative bands up to 10 km, but all had wide confidence intervals and the total number of cases within the 10-km radius circle was eight. The Stone test for trend was reported as *p* = 0.004. No systematic RF measurements have been made in the area, and the epidemiologic analyses are based on the simplistic proxy, distance from the source. The numbers of cases were small, especially for children, which precludes firm conclusions. For adults the results were inconsistent with the risk elevations largely confined to males.

### Discussion.

The research on community exposures to RFs and cancer gives a very weak test of the possibility of a relation. Diverse exposure sources, poorly estimated population exposures, small numbers of cases, and selective investigation in response to cluster concerns have resulted in a literature that is inconclusive. Despite apparent positive relations between proximity and leukemia incidence in some analyses (Hocking et al. 1996; Michelozzi et al. 2002), the results have not been consistent within or between studies and do not show relations to RF exposure levels. It seems to us that a prerequisite for a new generation of informative studies to emerge is the use of an RF meter.

Some of the concern about health risks from living near transmitters is directed toward symptoms such as fatigue, sleep disturbances, and frequent headaches. It may be tempting to address such issues in a cross-sectional study of people living near transmitters, in which subjects are asked to report their symptoms. Indeed, such studies have been done (Navarro et al. 2003; Santini et al. 2002, 2003). However, this is a design in which exposure is poorly characterized and reporting bias with respect to symptoms is of concern. Experimental designs easily overcome these biases and thus would be preferable, although they have their own limitations such as difficulty in practice in detecting effects present in a small percentage of a population or when the effect is not immediate. In these latter situations, an observational study would be the design of choice, but only if a design was found that avoided reporting bias.

## Review of Studies on Mobile Phone Use

Most studies of association between cancer and mobile phone use have evaluated the risk of brain tumors and acoustic neuromas (Table 7), although in a few instances the risks of other tumors have been explored. Also studies of symptoms in relation to mobile phone use have been conducted (Table 8). The first case–control study of brain tumors was conducted in Sweden (Hardell et al. 1999, 2000, 2001) and included adult cases diagnosed in two regions in Sweden between 1994 and 1996 and still alive, with two controls per case matched for region of residence. Details of intensity and duration of mobile phone use, preferred side (ear) of use, and whether phones were analog or digital, and handheld or hands-free, were gathered by postal questionnaire followed by telephone interview (Hardell et al. 1999). A total of 209 cases [about one-third of the malignant cases occurring in the study geographical area in the period (Ahlbom and Feychting 1999)] took part along with 425 controls (a reported 91% response rate—extraordinarily high for a contemporary population-based study). Originally no association of phone use with brain tumors was found (Hardell et al. 1999), although later reanalysis of side of use in relation to tumor site suggested a possible relationship (Hardell et al. 2001). A second larger study a few years later by the same authors (Hardell et al. 2002, 2003) was similar in design to the first. It involved 1,303 living cases (half of all brain tumors diagnosed 1997–2000) and their controls. Cumulative phone use for > 85 hr, 10 years before case diagnosis, gave ORs for brain tumors of 1.9 (95% CI, 1.1–3.2) and 3.0 (95% CI, 0.6–14.9), respectively, for analog and cordless phones, but ORs were not increased for digital phones. There was no adjustment for confounding variables. Ipsilateral use of analog phones was related to temporal tumors [OR = 2.5 (95% CI, 1.3–4.9)], and analog phone use was associated with acoustic neuroma [OR = 3.5 (95% CI, 1.8–6.8)] (Hardell et al. 2002, 2003).

Muscat et al. conducted two hospital-based case–control studies in the United States, one of malignant brain tumors (Muscat et al. 2000), the other of acoustic neuroma (Muscat et al. 2002), both using the same ascertainment and data collection procedures (Table 7). The first study included 469 cases of brain cancer (70% response rate) and 422 matched controls with a variety of malignant and benign conditions from the same hospitals (90% response rate). Information about mobile phone use was obtained by standard interview (of proxies for 9% of cases and 1% of controls). No increased risks were seen relating to frequency or duration of use, or for site or histologic subtype of brain cancer. An excess of brain cancer was found on the same side of the head as reported phone use among 41 cases with assessable data (*p* = 0.06), compared with a deficit on the side of mobile phone use for tumors specifically located in the temporal lobe (*p* = 0.33). In the acoustic neuroma study, 90 cases were compared with 86 controls, and no associations were seen with level or laterality of phone use.

In another U.S. hospital-based case–control study (Inskip et al. 2001), interview data were obtained from 782 cases with brain tumors (92% response rate; via proxies for 16% and 3% of glioma and acoustic neuroma patients, respectively) and 799 matched hospital controls with nonmalignant conditions (88% response; 3% by proxy). Results adjusted for potential confounders showed no association between cumulative use of mobile phones (mainly analog) and brain tumor overall or by histologic subtype or anatomical location.

Subscription records of national network providers were used to characterize mobile phone users in a Finnish case–control study (Auvinen et al. 2002). All people (398) diagnosed with brain tumors in 1996, ascertained from the National Cancer Registry, were matched with five controls per case drawn from the national population register (Table 7). The OR for brain tumors with ever-subscribed to phones was 2.1 (95% CI, 1.3–3.4) for analog phones and 1.0 for digital, and the OR for glioma was 1.5 (95% CI, 1.0–2.4) for any phone subscription. The average duration of subscription was 2–3 years for analog phones and less for digital. Adjusting for potential confounders did not alter results. No information was available about the frequency or duration of calls or about corporate subscriptions.

Of two cohort studies, an early U.S. study (Dreyer et al. 1999; Rothman et al. 1996) analyzed 1-year of follow-up of mortality in a cohort of 285,561 noncorporate users of mobile phones with at least two billing cycles from two U.S. carriers. Mortality was ascertained from the National Death Index. No relation was found between mortality from brain cancer and the use of handheld versus hands-free phones, based on only six cases. The overall mortality of the cohort was less that in the general population. The second cohort study was in Denmark (Johansen et al. 2002b) and included 420,095 private cellular network subscribers (80% of all subscribers), with average follow-up for analog and digital subscribers of 3.5 and 1.9 years, respectively. SIRs comparing cancer rates in phone users with national rates allowing for sex, age, and period showed no relation to risk of brain and nervous system cancers [SIR 0.95 (95% CI, 0.81–1.2)] and reduced risk of smoking-related cancers. Risks did not vary by age at, or time since, first subscription, phone type, or tumor location. Again, no information was available about the frequency or duration of calls or about corporate subscriptions.

Regarding other head and neck cancers, no association with parotid gland tumors (34 cases) was seen in the Finnish case–control study (Auvinen et al. 2002) or in the Danish cohort study (Johansen et al. 2002b). A mixed population and hospital-based case–control study of uveal melanoma (Stang et al. 2001) included 118 cases and 475 controls. Occupational exposure to mobile phones for several hours a day for ≥ 6 months assessed by interview gave an increased OR [4.2 (95% CI, 1.2–15)], reflecting the result in the hospital-based participants (OR = 10). There was no increased risk of uveal melanoma, however, in the Danish mobile phone user cohort (Johansen et al. 2002a). Finally, leukemia was assessed in both cohort studies, but no relation with phone use was found.

The first report from the multicenter Interphone study, a very large, international case–control study, has recently been published. This report from the Danish component focused on acoustic neuroma and was negative; however, the number of long-term users was small (Christensen et al. 2004).

Subjective symptoms, including tinnitus, headache, dizziness, fatigue, sensations of warmth, dysesthesia of the scalp, visual symptoms (e.g., flashes), memory loss, and sleep disturbance have been investigated in relation to mobile phone use (Chia et al. 2000; Oftedal et al. 2000; Sandstrom et al. 2001; details provided in Table 8). As discussed above in relation to transmitter studies, such research is highly susceptible to recall bias, and for completeness we have added Table 9, which includes experimental studies on mobile phone use and symptoms.

### Discussion.

Handheld mobile phones were not used regularly until the 1990s, so published studies at present can only assess relatively short lag periods before cancer manifestation. The relevant lag periods are unknown. Furthermore, even in the large Danish study (Johansen et al. 2002b), long-term (15 years) subscribers to analog phones comprised only a small proportion of users.

Another issue relates to choice of study population. No study populations to date have included children, yet children are increasingly heavy users of mobile phones and they are potentially highly susceptible to harmful effects (although some of these effects might not manifest until adulthood). So far, study populations have been ascertained from population registers in Nordic studies, hospital in-patients in U.S. case–control studies, and cellular network private subscribers in the two cohort studies and the Finnish study (Table 7). Although the population-based studies should have avoided the selection biases inherent in the hospital based studies, this was not so in population-based case–control studies of prevalent living cases with low participation rates (Hardell et al. 1999, 2002) because, inter alia, those with high-grade tumors tend to be excluded. Although rapid recruitment of incident brain tumor cases was facilitated in the hospital-based studies, loss due to death was still greater for malignant than benign tumors as reflected in differential proxy response rates by tumor type (Inskip et al. 2001), and there is a weakness in using hospital controls with a variety of conditions of unknown relationship to mobile phone use.

Differential recall of mobile phone use among those with and without a cerebral tumor in case–control studies is a major potential source of bias, exacerbated by differential timing of data collection from cases and controls in the hospital studies. Reporting bias is also likely because presence of a brain tumor may distort both memory and hearing and because the use of proxy respondents was more common for cases than controls. Relying on private cellular network subscription as a measure of mobile phone use would also have resulted in substantial misclassification because subscribers bear only a modest relation to users (Funch et al. 1996) and because corporate users were either excluded or included in the unexposed group. Until there is some objective measure of RF exposure, or at least validation of self-reported records, the validity of self-reported indices of phone use [e.g., average minutes of use per day (Hardell et al. 2002; Inskip et al. 2001) or minutes or hours per month as indicators of RF exposure] remains unknown.

Overall, although occasional significant associations between various types of brain tumors and analog mobile phone use have emerged (often seen after multiple testing), no single association has been consistently reported across population-based studies. The timing of epidemiologic studies and the lack of knowledge about actual RF exposure to the brain from mobile phone use to date (Ghandi et al. 1999) militate strongly against current ability to detect any true association. Thus current evidence is inconclusive regarding cancer risk after heavy RF exposure from mobile phones. Similarly, the studies of symptoms to date do not suggest that a single exposure to RFs from a mobile phone results in immediately identifiable symptoms, but there are no adequate data available about the symptomatic effects of mobile phone use, especially among people who claim hypersensitivity to RFs.

## General Conclusions and Recommendations

Results of epidemiologic studies to date give no consistent or convincing evidence of a causal relation between RF exposure and any adverse health effect. On the other hand, these studies have too many deficiencies to rule out an association.

A key concern across all studies is the quality of assessment of RF exposure, including the question of whether such exposure was present at all. Communication sources have increased greatly in recent years, and there is continuing change in the frequencies used and the variety of applications. Despite the rapid growth of new technologies using RFs, little is known about population exposure from these and other RF sources and even less about the relative importance of different sources. Certain studies that are currently under way have made serious attempts to improve exposure assessment, based on attempts to learn more about determinants of RF exposure levels. A key element in improving future studies would be the use of a meter that monitors individual exposure. In the absence of information on what biologic mechanism is relevant, if any, it is unclear what aspect of exposure needs to be captured in epidemiologic studies. Ideally, the dose needs to be assessed not just as external field intensity but also as cumulative exposure, as well as SAR, for specific anatomical sites.

The need for better exposure assessment is particularly strong in relation to transmitter studies, because the relation between distance and exposure is very weak. There is no point in conducting such studies unless it has been established that exposure levels vary substantially within the study area, and measurements of these RF levels are available. In the future, methods need to be developed to infer exposure based on some combination of knowledge regarding the sources of exposure, the levels of exposure, and location of people in relation to those sources, ideally informed by selective measurements.

Although the likelihood is low that fields emanating from base stations would create a health hazard because of their weakness, this possibility is nevertheless a concern for many people. To date no acceptable study on any outcome has been published on this. On the one hand, results from valid studies would be of value in relation to a social concern; on the other hand, it would be difficult to design and conduct a valid study, and there is no scientific point in conducting an invalid one.

Another general concern in mobile phone studies is that the lag periods that have been examined to date are necessarily short. The implication is that if a longer lag period is required for a health effect to occur, the effect could not be detected in these studies. Only in the few countries where mobile phones were introduced very early has it been possible to look at use ≥ 10 years ago. Much longer lag periods have been examined for occupational RF exposures, however. The published studies include some large occupational cohorts of good design and quality, except that there have been poor assessments of the degree of RF exposure, which render the results difficult to interpret.

Most research has focused on brain tumors and to some extent on leukemia. However, because the RF research questions are not driven by a specific biophysical hypothesis but rather by a general concern that there are unknown or misunderstood effects of RFs, studies on other health effects may be equally justified. Examples are eye diseases, neurodegenerative diseases, and cognitive function. Given the increase in new mobile phone technologies, it is essential to follow various possible health effects from the very beginning and for long periods, because such effects may be detected only after a long duration, because of the prolonged latency period of many chronic diseases. Thus, research is needed to address long-term exposure, as well as diseases other than those included in the ongoing case–control studies.

Another gap in the research is children. No study population to date has included children, with the exception of studies of people living near radio and TV antennas. Children are increasingly heavy users of mobile phones. They may be particularly susceptible to harmful effects (although there is no evidence of this), and they are likely to accumulate many years of exposure during their lives.

## Figures and Tables

**Table 1:** Cohort studies of risk of cancer in relation to occupational or hobby RF exposure: description of studies.

Reference	Occupational group	Sex	No. of subjects	Measure of exposure	Outcome
Milham 1988	Amateur radio operators	Male	67,829	Hobby title	Mortality
Garland et al. 1990	Navy personnel: electronics technicians, aviation electronics technicians, fire control technicians*^a^*	Male	Not stated	Job title	Incidence
Muhm 1992	Electromagnetic pulse test workers	Male	304	Job title	Mortality
Tynes et al. 1996	Radio and telegraph operators on merchant ships	Female	2,619	Measures in radio rooms of three ships	Incidence
Szmigielski 1996*^b^*	Military career personnel	Male	128,000 total,*^c^* 3,700 exposed*^c^*	Military health records; representative exposure levels given, based on measurements (no. not stated)	Incidence
Szmigielski et al. 2001	Military career personnel	Male	124,500 total, 3,900 exposed		
Lagorio et al. 1997	Dielectric RF heat sealer operators	Female	481	Unclear—stated that > 10 W/m^2^ frequently exceeded	Mortality
Morgan et al. 2000	Motorola employees	56% male, 44% female	195,775 total, 24,621 exposed	Job title, with expert assessment (not measured) of usual exposures	Mortality
Groves et al. 2002	Navy personnel with potential radar exposure	Male	40,581 total, 20,021 high exposure	Job title, plus expert assessment on potential for high exposure, and information on type and power of radar units	Mortality
Lilienfeld cited by Goldsmith 1995	U.S. embassy personnel	Males and females	Not stated	Moscow embassy service	Mortality

a We have extracted from the published article data on those jobs stated by Groves et al. (2002) to have greatest RF exposure.

b Not strictly a cohort study—there does not appear to be any follow-up; design appears to be calculation of annual rates, based on annual incidence and counts of employed population, and then averaging of these rates.

c Mean count each year”; presumably many but not all of the personnel will have been the same individuals from year to year of the study.

**Table 2:** Cohort studies of risk of cancer in relation to occupational RF exposure: results for brain tumor and leukemia.

		Brain tumor		Leukemia		
Reference	Type of analysis	No.	RR (95% CI)	No.	RR (95% CI)	Comment
Milham 1988	SMR, cohort vs. general	29	1.4 (0.9–2.0)	36	1.2 (0.9–1.7)	In a sample, 31% of subjects population worked in EMF-exposed occupations; analyses by license class, a proxy for duration of licensing, showed no consistent trend in risk.
Garland et al. 1990	SIR, cohort vs. general population					
	Electronics technician	—*a*		5	1.1 (0.4–2.5)	
	Aviation technician	—*a*		< 3	0.3 (0.0–1.9)	
	Fire control technician	—*a*		< 3	0.5 (0.0–2.5)	
Muhm 1992	SMR, cohort vs. general population,underlying cause	0	—	1	4.4 (0.1–24.3)	One of the leukemia cases may have been allocated to this work because of his leukemia.
	SMR, cohort vs. general population, mentioned cause	0	—	2	7.7 (0.9–28.0)	
	SIR, cohort vs. general population	—	—	2	5.4 (0.7–19.7)	
Tynes et al. 1996	SIR, cohort vs. general population	5	1.0 (0.3–2.3)	2	1.1 (0.1–4.1)	
Szmigielski 1996	Average crude incidence rate in exposed vs. average crude rate in unexposed	—*^a^*	1.9 (1.1–3.5)*^b^*	—*^a^*	7.7*^c^* (—*^a^*)	Poorly conducted and reported study; apparently more exposure data sources for cases than controls
Szmigielski et al. 2001		7	2.7 (*p* < 0.01)*^b^*	19	6.5 (*p* < 0.01)*^c^*	“Expected” rates in Szmigielski (1996) paper appear to be incorrect, according to the Royal Society of Canada (1999). Significant excesses were reported for several cancer sites not seen in other studies, and for cancer overall, suggesting possible bias. Analyses of risk in relation to exposure level were presented only for total cancer, not specific cancer sites.
Lagorio et al. 1997	SMR, cohort vs. general population	1	10	1	5	Potential confounding by chemical exposures; losses to follow-up treated as alive to end of study period
Morgan et al. 2000	SMR, exposed workers vs. general population	17	0.5 (0.2–1.1)	21	0.8 (0.4–1.4)	No duration–response trend
	Rate ratio exposed vs. unexposed in cohort, cumulative exposure					
	None	34	1.0	66	1.0	
	< Median	7	1.0 (0.4–2.2)	8	0.6 (0.3–1.3)	
	≥ Median	10	0.9 (0.4–1.9)	13	0.6 (0.3–1.0)	
Groves et al. 2002	SMR, overall cohort vs. general population	88	0.9 (0.7–1.1)	113	1.0 (0.8–1.2)	Significant increased risk for nonlymphocytic leukemia in high
	SMR, high exposure cohort vs. general population	37	0.7 (0.5–1.0)	69	1.1 (0.9–1.4)	exposure cohort, but only increased in one of three high-exposure
	Relative risk, exposed vs. unexposed in cohort	37/51	0.6 (0.4–1.0)	69/44	1.5 (1.0–2.2)	occupations
Lilienfeld cited by Goldsmith 1995*^d^*	Observed and expected, respectively, but source of latter unclear		Adults: 2/1.9 Children: 0/–		2/2.0 2/4.0	Data also presented for other U.S. embassies in Eastern Europe, but unclear whether they were exposed. Both children with brain tumors and one child with leukemia were dependents who lived outside the embassy.

Abbreviations: —, no data; CI, confidence interval; EMF, electromagnetic field; RR, relative risk.

a No data published; for Szmigielski (1996) it is implied that there were two to three brain tumors in the exposed group, in which case we imply that the 95% CI for brain tumor is incorrect.

b Nervous system.

c Calculated from data in the article.

d Study not published by Lilienfeld, and too little information given in précis in Goldsmith (1995) for understanding or evaluation of the methods. Small numbers of cancers, and several of the cancers occurred in persons who lived out of the embassy (i.e., presumably were in the embassy little of the time, especially children); breast cancer in employees: 2 observed, 0.5 expected; cancers of female genitalia: 4 observed, 0.8 expected; exposures estimated to range from 5 to 18 μW/cm^2^ (basis of estimate not stated).

**Table 3:** Case–control studies of risk of brain tumor and leukemia in relation to occupational RF exposure.

Reference	Sources of cases and controls*^a^*	Measure of exposure	Exposure data collection method	Mortality or incidence	No. of cases/controls	Type of analysis	Results [OR (95% CI)]	
							Brain tumor	Leukemia
Thomas et al. 1987	Cases: death certificates Controls: death certificates for deaths from other causes, except epilepsy, stroke, suicide, homicide	Job title and industry	Interview with relatives	Mortality	435/386	ORs vs. never occupationally exposed	1.6 (1.0–2.4)	—
Armstrong et al. 1994	Electrical utility workers (nested case–control)	Job exposure matrix based on 1 week meter measurements at 5–20 MHz*^c^* for > 1,000 workers, assessing exposure to pulsed electromagnetic fields	Company records	Incidence	84/325	ORs for ≥ median exposure	0.8 (0.5–1.5)*^b^*	—
					95/374	ORs for ≥ 90th percentile	1.9 (0.5–7.6)*^b^*	—
						OR for ≥ median exposure	—	0.7 (0.4–1.2)
						OR for ≥ 90th percentile	—	0.8 (0.2–3.4)
Grayson 1996	USAF (nested case–control)	Job title and reports of incidents of high exposure for each job title	Military records	Incidence	230/920	OR vs. never exposed	1.4 (1.0–1.9)	—

Abbreviations: —, no data; CI, confidence interval; ORs, odds ratios; USAF, U.S. Air Force.

a All studies restricted to men.

b Malignant brain tumors.

c It was later found that the meters also responded to fields of 150 and 300 MHz and to radio transmissions.

**Table 4:** Analyses of routinely collected data on brain tumor and leukemia risk in relation to occupational RF exposure.

References	Type of analysis	Exposed group*^a^*	Comparison cohort/control group	Mortality or incidence	Brain tumor		Leukemia	
					No.*^b^*	RR (95% CI)	No.*^b^*	RR (95% CI)
Wright et al. 1982	Proportional incidence	Radio and TV repairmen	All other cancers	Incidence	—		1	1.2 (—)
		Telephone linesmen			—		2	3.1 (—)
Calle and Savitz 1985	Proportional mortality	Radio and telegraph operators	All causes of death	Mortality	—		6	2.3 (—)
		Radio and TV repairmen			—		3	0.9 (—)
Lin et al. 1985	Case–control	Electric and telephone linemen, servicemen	Noncancer deaths	Mortality	27			—
Milham 1985	Proportional mortality	Radio and telegraph operators	All causes of deaths	Mortality	1	0.4 (—)	5	1.0 (—)
		Radio and TV repairmen			2	0.6 (—)	7	1.8 (—)
Pearce et al. 1989	Case–control	Radio and TV repairmen	All other cancers	Incidence	—		2	7.9 (2.2–28.1)
Tynes et al. 1996	Cohort	Radiofrequency-exposed occupations	Economically active males	Incidence	3	0.6 (0.1–1.8)	9	2.8 (1.3–5.4)

Abbreviations: —, no data published; CI, confidence interval; RR, relative risk.

a All studies are of males; exposure assessment for all is based solely on job title, with no measures of exposure.

b No. in exposed group.

**Table 5:** Summary of literature on RF exposure and reproductive health outcomes.

Outcome Semen parameters	Reference	Geographic setting	Population source and no.	Exposure and outcome
Semen parameters				
	Lancranjan et al. 1975	Romania	Microwave exposure (31) vs. controls (30)	Sperm count: 50 (exp), 60 (ctl) million/mL
				Percent motile: 36 (exp), 54 (ctl)
	Weyandt et al. 1996	United States	Military intelligence (20) vs. controls (30)	Sperm density: 13 (exp), 35 (ctl)
				Percent normal: 69 (exp), 73 (ctl)
				Percent motile: 32 (exp), 43 (ctl)
	Hjollund and Bonde 1997	Denmark	Military: missile operators (19), other (489)	Sperm density: 40 (exp), 62 (ctl)
				Percent immotile: 52 (exp), 33 (ctl)
				Percent normal: 61 (exp), 68 (ctl)
	Schrader et al. 1998	United States (Texas)	Military: radar operators (33), controls (103)	Sperm density: 29 (exp), 32 (ctl)
				Percent normal: 46 (exp), 42 (ctl)
				Percent motile: 46 (exp), 45 (ctl)
	Grajewski et al. 2000	United States (Maryland)	RF heater operators	Sperm density: 47 (exp), 45 (ctl)
				Sperm count: 73 (exp), 93 (ctl)
				Percent motile: 67 (exp), 52 (ctl)
				Normal morphology: 81 (exp), 79 (ctl)
Fertility				
	Larsen et al. 1991	Denmark	Physiotherapists (49), time to pregnancy > 6 months	TWA exposure and TTP > 6 months RR = 1.0, 0.8 (0.2–2.2), 1.7 (0.7–4.1)
Spontaneous abortion				
	Taskinen et al. 1990	Finland	Physiotherapists (204), spontaneous abortions	SAb ≤ 10
				Deep heat: 1.0, 1.3, 0.7
				Shortwaves: 1.0, 1.2, 0.7
				Microwaves 1.0, 0.7
				SAb > 10
				Deep heat: 1.0, 1.3, 2.6
				Shortwaves: 1.0, 2.5, 2.4
				Microwaves: 1.0, 2.4
	Larsen et al. 1991	Denmark	Physiotherapists (146), spontaneous abortions	TWA exposure and SAb: RR = 1.0, 1.0 (0.5–1.8), 1.4 (0.7–2.8)
	Ouellet-Hellstrom and Stewart 1993	United States	Female physical therapists (1,664), spontaneous abortions	Microwave diathermy exposures/month:
				RR = 1.0, 1.1 (0.8–1.4), 1.5 (1.0–2.2), 1.6 (1.0–2.6)
				Shortwave diathermy exposures/month:
				RR = 1.0, 1.2 (1.0–1.5), 1.1 (0.9–1.4), 0.9 (0.6–1.2)
Stillbirth				
	Larsen et al. 1991	Denmark	Physiotherapists (17), perinatal deaths	TWA exposure and perinatal death
				RR = 1.0, 1.5 (0.3–5.3), 2.9 (0.6–10.7)
Preterm birth				
	Larsen et al. 1991	Denmark	Physiotherapists (37 male, 45 female)	TWA exposure and preterm birth:
				Male: RR = 1.0, 1.4 (0.4–4.7), 3.2 (0.7–13.2)
				Female: RR = 1.0, 0.9 (0.4–2.1), 0.9 (0.3–2.8)
Low birth weight				
	Larsen et al. 1991	Denmark	Physiotherapists (15 male, 24 female)	TWA exposure and low birthweight:
				Male: RR = 1.0, 0.0, 5.9 (1.0–28.2)
				Female: RR = 1.0, 1.2 (0.4–3.3), 0.7 (0–3.2)
	Guberan et al. 1994	Switzerland	Physiotherapists (11 male, 14 female)	No association with shortwaves (RR not reported)
Birth defects				
	Logue et al. 1985	United States	Physical therapists (male), 192 birth defects	Observed: expected range “appears to be higher than expected”
	Taskinen et al. (1990)	Finland	Physiotherapists	Deep heat: 1.0, 2.4 (1.0–5.3), 0.9 (0.3–2.7)
			51 birth defects	Shortwaves: 1.0, 2.7 (1.2–6.1), 1.0 (0.3–3.1)
				Microwaves: 1.0, 0.5 (0.1–3.9)

Abbreviations: ctl, controls; exp, exposed; SAb, spontaneous abortions; TTP, time to pregnancy; TWA, time-weighted average.

**Table 6:** Summary of studies on transmitters and cancer.

Reference	Source of exposure	Comparison	End points	No. of cases	Results [OR (95% CI)]	Setting	Comments
Selvin et al. 1992	MW antenna	Internal	Childhood cancer	123	Random	San Francisco	Analysis of spatial data; no epidemiologic parameters
			Childhood leukemia	52	pattern		
Maskarinec et al. 1994	LF radio (23.4 kHz)	< 2.6 miles	Childhood leukemia	12	2.0 (0.06–8.3)	Hawaii	Case–control; SIR analysis on same cases: 2.09 (1.08–3.65)
Hocking et al. 1996	TV antenna	Inner/outer	All age leukemia		1.24 (1.09–1.40)	Northern Sydney	8–0.2 μW/cm^2^
			Childhood leukemia		1.58 (1.07–2.34)		
Dolk et al. 1997b	TV and FM radio	< 2 km	Adult leukemia	23	1.83 (1.22–2.74)	Sutton Coldfield	
Dolk et al. 1997a	TV and FM radio	< 2 km	Leukemia	79	0.97 (0.78–1.21)	All of Great Britain	
McKenzie et al. 1998	TV antennas	Continuous μW/cm^2^ model	Childhood leukemia			Sydney	Reanalysis of Hockings et al. (1996) with LGA analysis
Cooper et al. 2001	TV and FM radio	< 2 km	All age leukemia	20	1.32 (0.81–2.05)	Sutton	Reanalysis, more timely cancer data
			Childhood leukemia	1	1.13 (0.03–6.27)	Coldfield	
Michelozzi et al. 2002	Radio station	< 6 km	Childhood leukemia	8	2.2 (1.0–4.1)	Vatican	
			Adult leukemia	23	1.2 (0.8–1.8)		

Abbreviations: MW, microwave; LF, low frequency; LGA, local government area.

**Table 7:** Summary of studies of mobile phone use and risk of brain tumors.

Reference (study design)	Study population	Tumor type (nos. of cases/controls)	Exposure assessment	Mobile phone type;duration of use in controls	Mobile phone ever used [RR (95% CI)]
Hardell et al. 1999 (case–control)	Sweden	All tumors (209/425)	Recalled mobile phone use by questionnaire and interview	Mainly analog 450 or 900 MHz; 16% > 5 years	1.0 (0.7–1.4)*^a^*
	Cases: 20–80 years of age	Acoustic neuroma			0.8 (0.1–4.2)
	Controls: regional population registers, Uppsala-Orebro 1994–1996, Stockholm 1995–1996				
Muscat et al. 2000 (case–control)	United States: hospital inpatients, New York, Providence, Boston	Malignant brain tumor (469/422)	Recalled mobile phone use via interview	Mainly analog 800–900 MHz; 5% > 4 years	0.9 (0.6–1.2)
	Cases: 18–80 years,1994–1998				
	Controls: malignant and nonmalignant conditions				
Inskip et al. 2001 (case–control)	United States: hospital inpatients,	All tumors (782/799)	Recalled mobile phone use via interview	Mainly analog 800–900 MHz; 8% > 3 years	0.9 (0.7–1.1)
	Boston, Phoenix, Pittsburgh	Glioma (489/799)			1.0 (0.7–1.4)
	Cases: ≥ 18 years of age, 1994–1998	Meningioma (197/799)			0.8 (0.5–1.2)
	Controls: nonmalignant conditions	Acoustic neuroma (96/799)			0.8 (0.5–1.4)
Muscat et al. 2002 (case–control)	United States: hospital inpatients, New York	Acoustic neuroma (90/86)	Recalled mobile phone use via questionnaire	Mainly analog 800–900 MHz; 7% 3–6 years	0.9
	Cases: ≥ 18 years of age,1997–1999				
	Controls: nonmalignant conditions				
Auvinen et al. 2002 (case–control)	Finland	All tumors (398/1,986)	Duration of private cellular network subscription	Analog, average 2–3 years subscription; digital, average < 1 year subscription	1.3 (0.9–1.8)
	Cases: 20–69 years of age,1996	Glioma (198/989)			1.5 (1.0–2.4)
	Controls: national population register	Benign (129/643)			1.1 (0.5–2.4)
		Salivary gland (34/170)			1.3 (0.4–4.7)
Hardell et al. 2002 (case–control)	Sweden	All tumors (1,303/1,303)	Recalled mobile phone use via questionnaire	Analog 450 or 900 MHz, median 8 years	1.3 (1.0–1.6)*^a^*
	Cases: 20–80 years of age, 1997–2000				
	Controls: four regional population registers			Digital 1,900 MHz, median 3 years	1.0 (0.8–1.2)
Hardell et al. 2003 (case–control)		Acoustic neuroma (159/422)		Analog	3.5 (1.8–6.8)
				Digital	1.2 (0.7–2.2)
Dreyer et al. 1999 (cohort)	United States: subscribers of two large cellular networks, 1993	Malignant brain tumor (6)	Duration of subscription	Analog, 1 year follow-up	—
	Cases: ≥ 20 years of age, deaths 1994				—
Johansen et al. 2002 (cohort)	Denmark: private cellular network subscribers, 1982–1995	All tumors (154)	Duration of subscription	Analog 450 or 900 MHz or digital; up to 15 year follow-up	SIR 1.0 (0.8–1.1)
		Glioma (66)			0.9 (0.7–1.2)
	Cases: ≥ 18 years of age, 1982–1996	Menigioma (16)			0.9 (0.5–1.4)
Christensen et al. 2004	Denmark: population-based case–control	Acoustic neuroma (106); population controls (212)	—	—	0.90 (0.51–1.6)

a Analyzed with a 1-year lag period discounted.

**Table 8:** Summary of studies of mobile phone use and symptoms.

Reference (study design)	Study population	Analyses	Exposure assessment	Outcome assessment	Results
Oftedal et al. 2000 (cross-sectional)	Swedish and Norwegian mobile phone users, selected from network operator registers; included only people who used phone for job (*n* = 10,631)	1. Number of respondents with any symptom attributed to mobile phones	Self-completed questionnaire	Self-reported frequency of symptoms; patient considered to have symptom if occurred at least once per week	1. 13% of participants in Sweden and 31% in Norway reported at least one symptom in connection with use of a mobile phone; most common: warmth around ear; 22% of Norwegians and 7% of Swedes experienced symptom other than warmth.
		2. Number of respondents who had taken steps to reduce symptoms			2. 45% of people experiencing symptoms had taken steps to reduce them, such as reduced calling time, use of hands-free kit, changing side phone used.
Sandstrom et al. 2001 (cross-sectional)	Swedish and Norwegian mobile phone users, selected from network operator registers (*n* = 16,992)	1. Comparison of digital vs. analog mobile phone users	Self-completed questionnaire, variables; transmitter system, calling time per day and number of calls per day	Self-reported frequency of range of symptoms; participant considered to have symptoms if occurred at least once per week	1. OR among digital vs. analog phones: no increased risk for any symptoms; digital users at lower risk of warmth behind ear (OR = 0.64; 95% CI, 0.51–0.80) or on ear (OR = 0.68; 95% CI,0.53–0.86). Digital users in Sweden at lower risk of headaches (OR = 0.73; 95% CI, 0.56–0.95) and fatigue (OR = 0.80; 95% CI, 0.65–0.99).
		2. Trends with increasing time of phone usage			2. With increasing minutes of phone use there was an increased odds of reporting fatigue, headaches, warmth, burning, and tightness at least once per week.
Chia et al. 2000 (cross-sectional)	Random sample of 635 households in housing estate in Singapore; 808 respondents (response rate < 60%)	1. Prevalence ratio of headache in mobile phone users vs. non-users	Interviewer-administered questionnaire; purpose of study masked; classified as mobile phone user if used at least once per day	Questionnaire concerning nature and severity of “CNS symptoms” (headache, dizziness, warmth, tingling, visual disturbances); the frequency of headaches required before a respondent was classified as a headache sufferer was not specified	1. 45% mobile phone users; 3% experienced CNS problems; adjusted prevalence ratio for headache among users vs. non-users, 1.31 (95% CI, 1.00–1.70); no significant differences for any other symptoms.
		2. Association between minutes, phone use and headache			2. Significant positive trend for increasing time spent on the mobile phone and prevalence of headache (*p* = 0.04).

CNS, central nervous system.

**Table 9:** Summary of experimental studies of mobile phone use and symptoms.

Reference	Participants	Exposure	Protocol source	Symptoms reported	Results
Hietanen et al. 2002	20 volunteer subjects, mean age, 51 years for women and 47 years for men, all of whom classified themselves as hypersensitive to RFs	Analog phone, transmitting at 900 MHz; 900 and 1,800 MHz digital phones	Phones mounted near but not touching subjects ear; 3 or 4 experimental sessions lasting 30 min each, one of which was a sham exposure (random order)	Subjects asked to describe symptoms experienced during exposure; blood pressure, heart rate, and breathing frequency monitored; follow-up form used to measure symptoms over subsequent days	19/20 participants reported symptoms during the tests; compared with women during sham exposure, relative number of symptoms reported by female subjects during analog exposure was 0.82, digital 900 MHz, 0.79; digital 1,800 MHz, 0.72; among men, number of symptoms during any RF exposure situtation was 0.85 compared with sham exposure.
Koivisto et al. 2001	48 volunteers, students at University of Turku, Finland; mean age, 26 years	Digital 900 MHz phone	Two exposure sessions, one with mobile phone on and one with off; subjects blinded to whether phone was off or on; half of participants had phone on first and half off first	Questionnaire assessing symptoms administered in the beginning, middle, and end of session; subjects asked to rate strength of sensations on 4-point scale; symptoms assessed were dizziness, headache, fatigue, tingling, redness, warmth	There were no significant differences between mean values for subjective ratings between exposure on and exposure off situtations.

## References

[b1-ehp0112-001741] Ahlbom A, Feychting M (1999). Re: Use of cellular phones and the risk of brain tumours: a case-control study [Letter]. Int J Oncol.

[b2-ehp0112-001741] Anglesio L, Benedetto A, Bonino A, Colla D, Martire F, Saudino Fusette S (2001). Population exposure to electromagnetic fields generated by radio base stations: evaluation of the urban background by using provisional model and instrumental measurements. Radiat Prot Dosimetry.

[b3-ehp0112-001741] Armstrong B, Theriault G, Guenel P, Deadman J, Goldberg M, Heroux P (1994). Association between exposure to pulsed electromagnetic fields and cancer in electric utility workers in Quebec, Canada, and France. Am J Epidemiol.

[b4-ehp0112-001741] Auvinen A, Hietanen M, Luukkonen R, Koskela RS (2002). Brain tumors and salivary gland cancers among cellular telephone users. Epidemiology.

[b5-ehp0112-001741] Behin A, Hoang-Xuan K, Carpentier AF, Delattre JY (2003). Primary brain tumours in adults. Lancet.

[b6-ehp0112-001741] Calle EE, Savitz DA (1985). Leukemia in occupational groups with presumed exposure to electrical and magnetic fields. N Engl J Med.

[b7-ehp0112-001741] Cantor KP, Stewart PA, Brinton LA, Dosemeci M (1995). Occupational exposures and female breast cancer mortality in the United States. J Occup Environ Med.

[b8-ehp0112-001741] Chia SE, Chia HP, Tan JS (2000). Prevalence of headache among handheld cellular telephone users in Singapore: a community study. Environ Health Perspect.

[b9-ehp0112-001741] Christensen HC, Schuz J, Kosteljanetz M, Poulsen HS, Thomsen J, Johansen C (2004). Cellular telephone use and risk of acoustic neuroma. Am J Epidemiol.

[b10-ehp0112-001741] Cleary SF, Pasternack BS (1966). Lenticular changes in microwave workers. A statistical study. Arch Environ Health.

[b11-ehp0112-001741] Cleary SF, Pasternack BS, Beebe GW (1965). Cataract incidence in radar workers. Arch Environ Health.

[b12-ehp0112-001741] Cooper D, Hemmings K, Saunders P (2001). Re: “Cancer incidence near radio and television transmitters in Great Britain. I. Sutton Coldfield transmitter; II. All high power transmitters” [Letter]. Am J Epidemiol.

[b13-ehp0112-001741] COST281 (European Cooperation in the Field of Scientific and Technical Research.) 2001. Mobile Telecommunication Base Stations—Exposure to Electromagnetic Fields. Report of a Short Term Mission within COST 244bis. Available: http://www.cost281.org/activities/Short_term_mission.pdf [accessed 27 October 2004].

[b14-ehp0112-001741] Dahme M (1999). Residential RF exposures. Radiat Prot Dosimetry.

[b15-ehp0112-001741] Davis RL, Mostofi FK (1993). Cluster of testicular cancer in police officers exposed to hand-held radar. Am J Ind Med.

[b16-ehp0112-001741] Demers PA, Thomas DB, Rosenblatt KA, Jimenez LM, McTiernan A, Stalsberg H (1991). Occupational exposure to electromagnetic fields and breast cancer in men. Am J Epidemiol.

[b17-ehp0112-001741] Dimbylow PJ, Mann SM (1999). Characterization of energy deposition in the head from cellular phones. Radiat Prot Dosimetry.

[b18-ehp0112-001741] Dolk H, Elliott P, Shaddick G, Walls P, Thakrar B (1997a). Cancer incidence near radio and television transmitters in Great Britain. II. All high power transmitters. Am J Epidemiol.

[b19-ehp0112-001741] Dolk H, Shaddick G, Walls P, Grundy C, Thakrar B, Kleinschmidt I (1997b). Cancer incidence near radio and television transmitters in Great Britain. I. Sutton Coldfield transmitter. Am J Epidemiol.

[b20-ehp0112-001741] Dreyer NA, Loughlin JE, Rothman KJ (1999). Cause-specific mortality in cellular telephone users. JAMA.

[b21-ehp0112-001741] Funch DP, Rothman KJ, Loughlin JE, Dreyer NA (1996). Utility of telephone company records for epidemiologic studies of cellular telephones. Epidemiology.

[b22-ehp0112-001741] Garland FC, Shaw E, Gorham ED, Garland CF, White MR, Sinsheimer PJ (1990). Incidence of leukemia in occupations with potential electromagnetic field exposure in United States Navy personnel. Am J Epidemiol.

[b23-ehp0112-001741] Ghandi OP, Lazzi G, Tinniswood A, Yu QS (1999). Comparison of numerical and experimental methods for determination of SAR and radiation patterns of handheld wireless telephones. Bioelectromagnetics.

[b24-ehp0112-001741] Goldsmith JR (1995). Epidemiologic evidence of radiofrequency radiation (microwave) effects on health in military, broadcasting, and occupational studies. Int J Occup Environ Health.

[b25-ehp0112-001741] Goldstein LS, Kheifets L, van Deventer E, Repacholi M (2003). Comments on “Long-term exposure of Emicro-*Pim1* transgenic mice to 898.4 MHz microwaves does not increase lymphoma incidence” by Utteridge et al., Radiat. Res. 158, 357–364 (2002). Radiat Res.

[b26-ehp0112-001741] Grajewski B, Cox C, Schrader SM, Murray WE, Edwards RM, Turner TW (2000). Semen quality and hormone levels among radiofrequency heater operators. J Occup Environ Med.

[b27-ehp0112-001741] Grayson JK (1996). Radiation exposure, socioeconomic status, and brain tumor risk in the US Air Force: a nested case-control study. Am J Epidemiol.

[b28-ehp0112-001741] Groves FD, Page WF, Gridley G, Lisimaque L, Stewart PA, Tarone RE (2002). Cancer in Korean war navy technicians: mortality survey after 40 years. Am J Epidemiol.

[b29-ehp0112-001741] Hamburger S, Logue JN, Silverman PM (1983). Occupational exposure to non-ionizing radiation and an association with heart disease: an exploratory study. J Chronic Dis.

[b30-ehp0112-001741] Hardell L, Mild KH, Carlberg M (2002). Case-control study on the use of cellular and cordless phones and the risk for malignant brain tumours. Int J Radiat Biol.

[b31-ehp0112-001741] Hardell L, Mild KH, Carlberg M (2003). Further aspects on cellular and cordless telephones and brain tumours. Int J Oncol.

[b32-ehp0112-001741] Hardell L, Mild KH, Pahlson A, Hallquist A (2001). Ionizing radiation, cellular telephones and the risk for brain tumours. Eur J Cancer Prev.

[b33-ehp0112-001741] Hardell L, Nasman A, Pahlson A, Hallquist A (2000). Case-control study on radiology work, medical X-ray investigations, and use of cellular telephones as risk factors for brain tumors. MedGenMed.

[b34-ehp0112-001741] Hardell L, Nasman A, Pahlson A, Hallquist A, Hansson Mild K (1999). Use of cellular telephones and the risk for brain tumours: a case-control study. Int J Oncol.

[b35-ehp0112-001741] Hayes RB, Brown LM, Pottern LM, Gomez M, Kardaun JW, Hoover RN (1990). Occupation and risk for testicular cancer: a case-control study. Int J Epidemiol.

[b36-ehp0112-001741] Hietanen M, Hämäläinen AM, Husman T (2002). Hypersensitivity symptoms associated with exposure to cellular telephones: no causal link. Bioelectromagnetics.

[b37-ehp0112-001741] Hitchcock RT Patterson RM 1995. Radio-Frequency and ELF Electromagnetic Energies. A Handbook for Health Professionals. New York:Van Nostrand Reinhold.

[b38-ehp0112-001741] Hjollund NH, Bonde JP, Skotte J (1997). Semen analysis of personnel operating military radar equipment [Letter]. Reprod Toxicol.

[b39-ehp0112-001741] Hocking B, Gordon IR, Grain HL, Hatfield GE (1996). Cancer incidence and mortality and proximity to TV towers. Med J Aust.

[b40-ehp0112-001741] Hollows FC, Douglas JB (1984). Microwave cataract in radio-linemen and controls. Lancet.

[b41-ehp0112-001741] Holly EA, Aston DA, Ahn DK, Smith AH (1996). Intraocular melanoma linked to occupations and chemical exposures. Epidemiology.

[b42-ehp0112-001741] IEGMP 2000. Mobile Phones and Health. Chilton, Didcot, UK:Independent Expert Group on Mobile Phones. Available: http://www.iegmp.org.uk/report/text.htm [accessed 5 November 2004].

[b43-ehp0112-001741] Imaida K, Kuzutani K, Wang J, Fujiwara O, Ogiso T, Kato K (2001). Lack of promotion of 7,12-dimethylbenz[*a*]anthracene-initiated mouse skin carcinogenesis by 1.5 GHz electromagnetic near fields. Carcinogenesis.

[b44-ehp0112-001741] Inskip PD, Linet MS, Heineman EF (1995). Etiology of brain tumors in adults. Epidemiol Rev.

[b45-ehp0112-001741] Inskip PD, Tarone RE, Hatch EE, Wilcosky TC, Shapiro WR, Selker RG (2001). Cellular-telephone use and brain tumors. N Engl J Med.

[b46-ehp0112-001741] Jauchem JR (1997). Exposure to extremely-low-frequency electromagnetic fields and radiofrequency radiation: cardiovascular effects in humans. Int Arch Occup Environ Health.

[b47-ehp0112-001741] Johansen C, Boice JD, McLaughlin JK, Christensen HC, Olsen JH (2002a). Mobile phones and malignant melanoma of the eye. Br J Cancer.

[b48-ehp0112-001741] Johansen C, Boice JD, McLaughlin JK, Olsen JH (2002b). Cellular telephones and cancer—a nationwide cohort study in Denmark. J Natl Cancer Inst.

[b49-ehp0112-001741] Koivisto M, Haarala C, Krause CM, Revonsuo A, Laine M, Hämäläinen H (2001). GSM phone signal does not produce subjective symptoms. Bioelectromagnetics.

[b50-ehp0112-001741] Krewski D, Byus CV, Glickman BW, Lotz WG, Mandeville R, McBride ML (2001). Potential health risks of radio-frequency fields from wireless telecommunication devices. J Toxicol Environ Health B Crit Rev.

[b51-ehp0112-001741] Lagorio S, Rossi S, Vecchia P, De Santis M, Bastianini L, Fusilli M (1997). Mortality of plastic-ware workers exposed to radiofrequencies. Bioelectromagnetics.

[b52-ehp0112-001741] Lancranjan I, Maicanescu M, Rafaila E, Klepsch I, Popescu HI (1975). Gonadic function in workmen with long-term exposure to microwaves. Health Phys.

[b53-ehp0112-001741] Larsen AI (1991). Congenital malformations and exposure to high-frequency electromagnetic radiation among Danish physiotherapists. Scand J Work Environ Health.

[b54-ehp0112-001741] Larsen AI, Olsen J, Svane O (1991). Gender-specific reproductive outcome and exposure to high-frequency electromagnetic radiation among physiotherapists. Scand J Work Environ Health.

[b55-ehp0112-001741] Larsen AI, Skotte J (1991). Can exposure to electromagnetic radiation in diathermy operators be estimated from interview data? A pilot study. Am J Ind Med.

[b56-ehp0112-001741] Lin RS, Dischinger PC, Conde J, Farrell KP Occupational exposure to electromagnetic fields and the occurrence of brain tumors. An analysis of possible associations. J Occup Med.

[b57-ehp0112-001741] Logue JN, Hamburger S, Silverman PM, Chiacchierini RP Congenital anomalies and paternal occupational exposure to shortwave, microwave, infrared, and acoustic radiation. J Occup Med.

[b58-ehp0112-001741] Mantiply ED, Pohl KR, Poppell SW, Murphy JA (1997). Summary of measured radiofrequency electric and magnetic fields (10 kHz to 30 GHz) in the general and work environment. Bioelectromagnetics.

[b59-ehp0112-001741] Maskarinec G, Cooper J, Swygert L (1994). Investigation of increased incidence in childhood leukemia near radio towers in Hawaii: preliminary observations. J Environ Pathol Toxicol Oncol.

[b60-ehp0112-001741] Mason PA, Walters TJ, DiGiovanni J, Beason CW, Jauchem JR, Dick EJ (2001). Lack of effect of 94 GHz radio frequency radiation exposure in an animal model of skin carcinogenesis. Carcinogenesis.

[b61-ehp0112-001741] McKenzie DR, Yin Y, Morrell S (1998). Childhood incidence of acute lymphoblastic leukaemia and exposure to broadcast radiation in Sydney—a second look. Aust NZ J Public Health.

[b62-ehp0112-001741] Michelozzi P, Capon A, Kirchmayer U, Forastiere F, Biggeri A, Barca A (2002). Adult and childhood leukemia near a high-power radio station in Rome, Italy. Am J Epidemiol.

[b63-ehp0112-001741] Milham S (1985). Silent keys: leukaemia mortality in amateur radio operators [Letter]. Lancet.

[b64-ehp0112-001741] Milham S (1988). Increased mortality in amateur radio operators due to lymphatic and hematopoietic malignancies. Am J Epidemiol.

[b65-ehp0112-001741] Morgan RW, Kelsh MA, Zhao K, Exuzides KA, Heringer S, Negrete W (2000). Radiofrequency exposure and mortality from cancer of the brain and lymphatic/hematopoietic systems. Epidemiology.

[b66-ehp0112-001741] Moulder JE, Erdreich LS, Malyapa RS, Merritt J, Pickard WF, Vijayalaxmi (1999). Cell phones and cancer: what is the evidence for a connection?. Radiat Res.

[b67-ehp0112-001741] Muhm JM (1992). Mortality investigation of workers in an electromagnetic pulse test program. J Occup Med.

[b68-ehp0112-001741] Muscat JE, Malkin MG, Shore RE, Thompson S, Neugut AI, Stellman SD (2002). Handheld cellular telephones and risk of acoustic neuroma. Neurology.

[b69-ehp0112-001741] Muscat JE, Malkin MG, Thompson S, Shore RE, Stellman SD, McRee D (2000). Handheld cellular telephone use and risk of brain cancer. JAMA.

[b70-ehp0112-001741] Navarro EA, Segura J, Portolés M, Gómez-Perretta de Mateo C (2003). The microwave syndrome: a preliminary study in Spain. Electromagn Biol Med.

[b71-ehp0112-001741] NRPB 2003. Health Effects from Radiofrequency Electromagnetic Fields. Report of an Independent Advisory Group on Non-ionising Radiation. Chilton, Didcot, UK:National Radiation Protection Board. Available: http://www.nrpb.org/publications/documents_of_nrpb/pdfs/doc_14_2.pdf [accessed 28 October 2004].

[b72-ehp0112-001741] Oftedal G, Wilen J, Sandstrom M, Mild KH (2000). Symptoms experienced in connection with mobile phone use. Occup Med (Lond).

[b73-ehp0112-001741] Ouellet-Hellstrom R, Stewart WF (1993). Miscarriages among female physical therapists who report using radio- and microwave-frequency electromagnetic radiation. Am J Epidemiol.

[b74-ehp0112-001741] Pearce N, Reif J, Fraser J Case-control studies of cancer in New Zealand electrical workers. Int J Epidemiol.

[b75-ehp0112-001741] Petridou E Trichopoulos D 2002. Leukemias. In: Textbook of Cancer Epidemiology (Adami A, Trichopoulos D, Hunter D, eds). Oxford, UK:Oxford University Press, 556–572.

[b76-ehp0112-001741] Repacholi MH, Basten A, Gebski V, Noonan D, Finnie J, Harris AW (1997). Lymphomas in E mu-*Pim1* transgenic mice exposed to pulsed 900 MHz electromagnetic fields. Radiat Res.

[b77-ehp0112-001741] Robinette CD, Silverman C, Jablon S (1980). Effects upon health of occupational exposure to microwave radiation (radar). Am J Epidemiol.

[b78-ehp0112-001741] Rothman KJ, Loughlin JE, Funch DP, Dreyer NA (1996). Overall mortality of cellular telephone customers. Epidemiology.

[b79-ehp0112-001741] Royal Society of Canada 1999. A Review of the Potential Health Risks of Radiofrequency Fields from Wireless Telecommunication Devices. Ottawa, Ontario:Royal Society of Canada. Available: http://www.rsc.ca/english/RFreport.pdf [accessed 4 November 2004].

[b80-ehp0112-001741] Sandstrom M, Wilen J, Oftedal G, Hansson Mild K (2001). Mobile phone use and subjective symptoms. Comparison of symptoms experienced by users of analogue and digital mobile phones. Occup Med (Lond).

[b81-ehp0112-001741] Santini R, Santini P, Danze JM, Le Ruz P, Seigne M (2002). Investigation on the health of people living near mobile telephone relay stations: I/Incidence according to distance and sex. Pathol Biol (Paris).

[b82-ehp0112-001741] Santini R, Santini P, Danze JM, Le Ruz P, Seigne M (2003). Symptoms experienced by people in vicinity of base stations: II/Incidences of age, duration of exposure, location of subjects in relation to the antennas and other electromagnetic factors. Pathol Biol (Paris).

[b83-ehp0112-001741] Savitz DA, Dufort V, Armstrong B, Theriault G (1997). Lung cancer in relation to employment in the electrical utility industry and exposure to magnetic fields. Occup Environ Med.

[b84-ehp0112-001741] Schrader SM, Langford RE, Turner TW, Breitenstein MJ, Clark JC, Jenkins BL (1998). Reproductive function in relation to duty assignments among military personnel. Reprod Toxicol.

[b85-ehp0112-001741] Schüz J, Mann S (2000). A discussion of potential exposure metrics for use in epidemiological studies on human exposure to radiowaves from mobile phone base stations. J Expo Anal Environ Epidemiol.

[b86-ehp0112-001741] Selvin S, Schulman J, Merrill DW (1992). Distance and risk measures for the analysis of spatial data: a study of childhood cancers. Soc Sci Med.

[b87-ehp0112-001741] Shacklett DE, Tredici TJ, Epstein DL (1975). Evaluation of possible microwave-induced lens changes in the United States Air Force. Aviat Space Environ Med.

[b88-ehp0112-001741] Silvi AM, Zari A, Licitra G (2001). Assessment of the temporal trend of the exposure of people to electromagnetic fields produced by base stations for mobile telephones. Radiat Prot Dosimetry.

[b89-ehp0112-001741] Stang A, Anastassiou G, Ahrens W, Bromen K, Bornfeld N, Jockel KH (2001). The possible role of radiofrequency radiation in the development of uveal melanoma. Epidemiology.

[b90-ehp0112-001741] Swerdlow AJ (1999). Measurement of radiofrequency radiation exposure in epidemiological studies. Radiat Prot Dosimetry.

[b91-ehp0112-001741] Szmigielski S (1996). Cancer morbidity in subjects occupationally exposed to high frequency (radiofrequency and microwave) electromagnetic radiation. Sci Total Environ.

[b92-ehp0112-001741] Szmigielski S, Sobiczewska E, Kubacki R (2001). Carcinogenic potency of microwave radiation: overview of the problem and results of epidemiological studies on Polish military personnel. Eur J Oncol.

[b93-ehp0112-001741] Taskinen H, Kyyronen P, Hemminki K (1990). Effects of ultrasound, shortwaves, and physical exertion on pregnancy outcome in physiotherapists. J Epidemiol Community Health.

[b94-ehp0112-001741] Thomas TL, Stolley PD, Stemhagen A, Fontham ET, Bleecker ML, Stewart PA (1987). Brain tumor mortality risk among men with electrical and electronics jobs: a case-control study. J Natl Cancer Inst.

[b95-ehp0112-001741] Tynes T, Hannevik M, Andersen A, Vistnes AI, Haldorsen T (1996). Incidence of breast cancer in Norwegian female radio and telegraph operators. Cancer Causes Control.

[b96-ehp0112-001741] Utteridge TD, Gebski V, Finnie JW, Vernon-Roberts B, Kuchel TR (2002). Long-term exposure of E-mu-*Pim1* transgenic mice to 898.4 MHz microwaves does not increase lymphoma incidence. Radiat Res.

[b97-ehp0112-001741] Weyandt TB, Schrader SM, Turner TW, Simon SD (1996). Semen analysis of military personnel associated with military duty assignments. Reprod Toxicol.

[b98-ehp0112-001741] Wright WE, Peters JM, Mack TM Leukaemia in workers exposed to electrical and magnetic fields. Lancet.

